# Evaluation of whole-body and segmental bioimpedance between normal weight obese and normal weight non-obese women

**DOI:** 10.1080/15502783.2025.2550193

**Published:** 2025-08-30

**Authors:** Callie L. Unrein, Kaitlyn T. Ramey, Katelynn T. Persaud, Sarah J. Rhoades, Katie R. Hirsch

**Affiliations:** University of South Carolina, Department of Exercise Science, Arnold School of Public Health, Columbia, SC, USA

**Keywords:** Metabolic risk, body composition, BMI, fat mass

## Abstract

**Introduction:**

Normal weight obesity (NWO), defined by a BMI ≤ 24.9 kg/m^2^ and %BF ≥ 30%, is a high-risk phenotype linked to elevated metabolic risk and is more prevalent in females. Compared to normal weight non-obesity (NW), NWO may present with greater visceral fat, lower fat-free mass (FFM), and reduced muscle quality, particularly in the legs – differences not captured by BMI or %BF alone. Bioelectrical impedance analysis (BIA) provides raw parameters (impedance [Z], resistance [R], reactance [Xc], phase angle [PhA]) that may offer further insight into tissue characteristics. Lower R is associated with increased FFM and reduced muscle mass, while higher Xc and PhA reflect better cellular integrity. This study evaluated whole-body (WB) and segmental (trunk [Trk], right leg [RL]) bioimpedance differences between NWO and NW women.

**Methods:**

Six NWO women [BMI(kg/m^2^): 22.5 ± 0.9; %BF: 31.4 ± 0.8; FFM(kg): 43.8 ± 2.0; VAT(g): 170.3 ± 57.1] and six NW women [BMI(kg/m^2^): 20.6 ± 0.7; %BF: 23.3 ± 1.9; FFM(kg): 45.7 ± 4.9; VAT(g): 146.7 ± 38.9] completed a single visit during the low hormone phase (days 1–7 after menses). Body composition was assessed via DXA, and bioimpedance was measured at 50 kHz using BIA. Independent sample t-tests and Cohen’s d were used to evaluate group differences in demographics, body composition, and bioimpedance outcomes (Z, R, Xc, PhA) across WB, Trk, and RL segments.

**Results:**

NWO had significantly higher BMI [Mean difference (∆) ± SE: 2.0 ± 0.5 kg/m^2^, *p* = 0.002] and %BF (∆: 8.1 ± 0.9%, *p* < 0.001) compared to NW, with %BF differences observed across all segments: RL (∆: 8.3 ± 1.9%, *p* = 0.002), and Trk (∆: 7.9 ± 1.6%, *p* < 0.001). While not statistically significant, NWO exhibited lower FFM(kg): [WB (∆: −1.9 ± 2.2, *p* = 0.388, d=–0.521), RL (∆: −0.5 ± 0.9, *p* = 0.567, d=–0.342), and Trk (∆: −2.4 ± 2.5, *p* = 0.358, d=–0.556)], as well as higher VAT(g): (∆: 23.7 ± 28.2, *p* = 0.420, d = 0.485). There were no significant differences in bioimpedance outcomes between NWO and NW. However, NWO tended to have higher bioimpedance across all segments, with moderately large effects for Z, R, and Xc [WB (d = 0.708–0.982), RL (d = 0.817–0.819), Trk (d = 0.923–1.031)] and small to moderate effects for PhA (d = 0.031–0.401).

**Conclusion:**

Despite a normal BMI, NWO women had greater adiposity and lower FFM, highlighting BMI’s limitations in assessing metabolic risk. Higher R likely reflects greater fat mass, while elevated Xc and PhA may reflect greater total mass rather than cellular integrity. Given BIA’s limitations in isolating muscle quality, pairing it with another noninvasive tool, such as ultrasound, may provide further insight.

